# What Contribution Did Economic Evidence Make to the Adoption of Universal Newborn Hearing Screening Policies in the United States?

**DOI:** 10.3390/ijns4030025

**Published:** 2018-07-20

**Authors:** Scott D. Grosse, Craig A. Mason, Marcus Gaffney, Vickie Thomson, Karl R. White

**Affiliations:** 1 National Center on Birth Defects and Developmental Disabilities, Centers for Disease Control and Prevention, 4770 Buford Highway NE, Mail Stop E-87, Atlanta, GA 30341, USA; 2 College of Education and Human Development, University of Maine, Orono, ME 00469, USA; 3 Department of Otolaryngology, University of Colorado Denver, Denver, CO 80045, USA; 4 National Center for Hearing Assessment and Management (NCHAM), Utah State University, Logan, UT 84322, USA; 5 Department of Psychology, Utah State University, Logan, UT 84322, USA

**Keywords:** cost-effectiveness, cost analysis, EHDI, neonatal screening, hearing screening

## Abstract

Universal newborn hearing screening (UNHS), when accompanied by timely access to intervention services, can improve language outcomes for children born deaf or hard of hearing (D/HH) and result in economic benefits to society. Early Hearing Detection and Intervention (EHDI) programs promote UNHS and using information systems support access to follow-up diagnostic and early intervention services so that infants can be screened no later than 1 month of age, with those who do not pass their screen receiving diagnostic evaluation no later than 3 months of age, and those with diagnosed hearing loss receiving intervention services no later than 6 months of age. In this paper, we first document the rapid roll-out of UNHS/EHDI policies and programs at the national and state/territorial levels in the United States between 1997 and 2005. We then review cost analyses and economic arguments that were made in advancing those policies in the United States. Finally, we examine evidence on language and educational outcomes that pertain to the economic benefits of UNHS/EHDI. In conclusion, although formal cost-effectiveness analyses do not appear to have played a decisive role, informal economic assessments of costs and benefits appear to have contributed to the adoption of UNHS policies in the United States.

## 1. Introduction

Permanent congenital hearing loss (PCHL), which affects 1.5–3.0 per 1000 infants, places children who are born deaf or hard of hearing (D/HH) at risk for delayed language development. In the absence of universal newborn hearing screening (UNHS), many children are not diagnosed with PCHL until 2 years of age or later [1], at which point delays in language development are more difficult to remediate [2,3]. For example, among 173 children in one metropolitan area who were aged 3–10 years during 1991–1993 and had moderate to profound PCHL, the mean age at first diagnosis was 2.9 years and just 13 (7.5%) had been identified with hearing loss or deafness prior to 1 year of age [4]. 

UNHS aims to ensure that all infants who are D/HH are diagnosed before 3 months of age and begin receiving interventions aimed at supporting language development before 6 months of age. Prior to the implementation of UNHS policies circa 2000, the recommended practice in the United States (US) was targeted or selective screening of newborns based on risk factors, such as stays in a neonatal intensive care unit (NICU) or a family history of deafness. However, targeted newborn hearing screening was never widely implemented in the United States, and when implemented in the United Kingdom, was not as effective as expected in identifying infants with PCHL [5,6]. 

During the late 1980s and early 1990s, new technologies made the routine screening of newborn infants for hearing loss or deafness by hospital staff feasible [7,8]. That development was followed by the implementation beginning in 1990 of population-level pilot UNHS programs in Denmark [9] and the United States [8], and by a large randomized trial in the United Kingdom (UK) conducted during 1993–1996 [10,11]. In the United States, consensus was soon reached that UNHS was feasible, affordable, and warranted on the basis of evidence from observational studies of better language outcomes with earlier identification [12]. By the end of the 1990s, professional organizations and federal agencies recognized the need for UNHS to achieve timely identification, and action was initiated at both the state and federal levels. In addition, those stakeholders realized that policy support was needed to ensure access to both screening and diagnostic testing for early identification, followed by intervention services (e.g., training in sign language, hearing amplification services, speech language services, etc.). To support early identification and improved outcomes, Early Hearing Detection and Intervention (EHDI) partners developed national guidelines that called for infants to be screened no later than 1 month of age, with those who do not pass their screen receiving diagnostic evaluation no later than 3 months of age, and that infants identified with hearing loss or deafness receive intervention services no later than 6 months of age [7,8,13]. These are referred to as the 1-3-6 goals.

This report first describes the evolution of EHDI policies and programs in the US, followed by summaries of cost assessments of hospital-based UNHS programs and economic assessments of the expected costs and benefits of UNHS/EHDI policies. Those are followed by a discussion of how economic assessments, including cost analyses, informed those policy development processes. That discussion distinguishes formal economic evaluations, such as cost-effectiveness analyses, and informal or partial economic assessments, including cost assessments and comparisons of expected costs with simple calculations of potential benefits. 

### 1.1. Evolution of EHDI Policies in the United States

During 1990–1992, two states (Rhode Island and Hawaii) began UNHS through state authorizing legislation, dedicated funding from the US Health Resources and Services Administration (HRSA) Maternal and Child Health Bureau (MCHB), and technical assistance from the National Center for Hearing Assessment and Management (NCHAM) at Utah State University [14,15]. In March 1993, a National Institutes of Health (NIH) Consensus Development Conference on Early Identification of Hearing Impairment called for UNHS prior to 3 months of age in combination with comprehensive intervention and management programs. This recommendation was supported by a 1994 Position Statement of the Joint Committee on Infant Hearing (JCIH), which was originally convened in 1969 by the American Speech Language Hearing Association (ASHA), the then American Academy of Ophthalmology and Otolaryngology (AAOO), and the American Academy of Pediatrics (AAP). In September, 1997, academic experts convened at a National Institute on Deafness and Other Communication Disorders (NIDCD) Workshop on UNHS and recommended that hearing screening be conducted before 3 months to ensure that infants identified with hearing loss or deafness could begin intervention before 6 months of age. (The report of the workshop is available at: https://www.nidcd.nih.gov/research/workshops/working-group-early-identification-hearing-impairment/1997).

The first concrete steps to implementing UNHS as official policy throughout the United States came in October 1993, following the NIH Consensus Development Conference. HRSA awarded funding for 3 years to NCHAM at Utah State University for the establishment of a Technical Assistance Consortium for Universal Newborn Auditory Screening. Subsequently, in 1996 HRSA awarded a grant to the University of Colorado to establish the Marion Downs National Center for Infant Hearing to support 17 states in the development of UNHS programs [7]. In addition, ASHA developed draft model legislation for states that was released in 1997. Four states adopted UNHS legislation during 1997, followed by three more states in 1998. In 1999, 14 additional states adopted similar legislation and the AAP formally endorsed UNHS [16]. 

Colorado was one of the first US states to take action to implement UNHS. A voluntary Colorado Newborn Hearing Screening (CNHS) Project established in 1992 recommended that hospitals screen newborns for hearing loss or deafness, although no funding was provided [17]. By 1997, 26 of 56 birthing hospitals in the state were participating in the CNHS project, and more than 60% of infants born in the state were screened [18]. In 1997, the Colorado legislature overwhelmingly passed legislation that required that all hospitals offer newborn hearing screening. It also required that midwives offer education on how parents of newborn infants delivered outside of a hospital could obtain newborn hearing screening. In addition, the legislation authorized the creation of an advisory committee and programmatic support from the Colorado Department of Public Health and the Environment [18].

In March 1999, Representative James Walsh of the US House of Representatives introduced the Newborn Infant Hearing Screening and Intervention Act, which was incorporated into Title VI of the Labor, Health and Human Services, and Education Appropriations Act of 1999 and signed into law. That legislative language, which was also included in the Children’s Health Act of 2000, provided funding to both HRSA and the Centers for Disease Control and Prevention (CDC). Two other landmark endorsements of UNHS came in 2000. First, the US Department of Health and Human Services (HHS) in January 2000 released national Healthy People 2010 objectives, three of which corresponded to the EHDI 1-3-6 plan goals: “28-11a. Newborns receiving hearing screening before age 1 month; 28-11b. Infants with possible hearing loss receiving hearing evaluation before age 3 months; and 28-11c. Infants with hearing loss receiving intervention services before age 6 months” [8,19]. Second, the JCIH issued a Position Statement in support of UNHS [20]. 

Beginning in 2000, states and territories became eligible to receive funds from HRSA and CDC to establish programs to support UNHS and monitor the receipt of screening, diagnostic, and intervention services. In addition, HRSA awarded a grant to Utah State University to operate a National Technical Assistance System for EHDI programs [7]. At about the same time, an EHDI program was established within CDC’s National Center on Birth Defects and Developmental Disabilities (NCBDDD) to provide both funding and technical assistance to support the development and implementation of data management and tracking systems within state EHDI/UNHS programs. CDC was also tasked with developing national data standards and tracking progress towards achievement of EHDI goals. Between 2000 and 2003, the number of states that reported hearing screening data and had adopted UNHS legislation increased from 16 to 36 and the number with fully implemented legislation rose from 6 to 21 [21]. By the end of 2001, all 50 states had established an EHDI program to support implementation of UNHS and the 1-3-6 EHDI goals [7]. 

In 2005, HHS adopted a recommended uniform screening panel (RUSP), as recommended by the Secretary’s Advisory Committee on Heritable Disorders in Newborns and Children [22]. The RUSP lists conditions that all states and other jurisdictions are encouraged to include in their newborn screening policies, which since the beginning has included “hearing loss.” (https://www.hrsa.gov/advisory-committees/heritable-disorders/rusp/index.html). 

The US Preventive Services Task Force (USPSTF) is a voluntary, non-governmental advisory body of primary care clinicians which is staffed by the Agency for Healthcare Research and Quality and provides evidence-based recommendations on clinical preventive services for primary care. The USPSTF concluded in 2001, based on a commissioned systematic evidence review [23], that there was insufficient evidence to recommend UNHS but sufficient evidence to recommend selective, risk-based infant hearing screening [24]. The evidence review assumed that among infants who had been in a NICU “the risk of moderate-to-severe PHL is 10 to 20 times higher than in the general population” [23]. However, the two largest US studies in their Table 1 had NICU/non-NICU case ratios of 7.7 [25] and 8.9 [26]. The percentage of infants whose hearing loss or deafness would be detected through selective screening of high-risk infants, if such screening were to be uniformly implemented, may therefore have been overestimated in the USPSTF review. In marked contrast to its approach to selective screening, the USPSTF review set a high evidentiary bar for UNHS. For example, the authors of the review dismissed evidence from a Colorado study comparing outcomes among infants born at hospitals with and without UNHS [18] as being of “poor” quality because it ostensibly compared screened and unscreened cohorts [23]. In fact, the study used an “intent-to-treat” analysis in which infants were characterized by the screening protocol of the birth hospital, not whether the infants were themselves screened [18]. As late as 2005, supporters continued to justify the USPSTF refusal to recommend UNHS, arguing that the evidence for the “idea that late diagnosis produces lasting speech and language problems” was “relatively weak” [27]. However, that same argument undermined the logic of the USPSTF endorsement of selective hearing screening. If there was insufficient evidence that timely diagnosis of hearing loss or deafness produces lasting benefits, what was the logical basis to recommend selective screening?

In 2008, a new USPSTF evidence review concluded that there was sufficient evidence of improved language outcomes [28] and a recommendation in favor of UNHS was finally issued by the USPSTF [29]. The new evidence was provided by a 2006 report of data from the UK Hearing Outcomes Project, which compiled data from a controlled trial together with an observational screening study of UNHS in different English health districts. That study reported statistically significantly improvements in language development among school-aged children who were D/HH in association with UNHS [30]. The USPSTF recommendation of UNHS came with a B grade, indicating “high certainty that the net benefit is moderate or there is moderate certainty that the net benefit is moderate to substantial” [29]. All preventive services which are supported by an A or B recommendation from the USPSTF are required by federal law to be covered by most payers at no cost to the consumer [31].

### 1.2. Trends in UNHS and EHDI Objectives in the United States

White reviewed the early history of EHDI in the United States [7]. According to NCHAM, the estimated percentage of US infants who were screened for hearing loss prior to hospital discharge consistently increased from approximately 10% in 1996 to 20% in 1998, 30% in 1999, 50% in 2000, and 65% in 2001.

From 1999 through 2004, state estimates of the percentages of infants screened for hearing loss were voluntarily collected and shared with CDC by the Directors of Speech and Hearing Programs in State Health and Welfare Agencies (DSHPSHWA) through an annual survey of state EHDI programs. Using those data, Green et al. found that during 2000 the percentage of infants screened differed markedly across states, depending on whether UNHS legislation had been enacted and implemented in that state [21]. In 18 states without UNHS legislation, 53% of infants were reported screened, compared with 95% in states with fully implemented UNHS legislation. By 2003, the gap had diminished to just 5 percentage points: 90% in 13 states without legislation and 95% in 21 states with fully implemented legislation. 

Since 2005, CDC has annually collected data from state and territorial EHDI programs through the Hearing Screening and Follow-up Survey (HSFS) about the receipt of screening, diagnostic, and intervention services among infants. In 2005, HSFS data pooled from 48 jurisdictions indicated that 94% of infants were screened for hearing loss [32], and by 2014 that figure had risen to 98% [33] (https://www.cdc.gov/ncbddd/hearingloss/ehdi-data.html).

## 2. Review of US Estimates of UNHS Costs and Cost Savings

Economic evaluations vary in scope and purpose. Partial economic evaluations include assessments of either the cost of delivering a service or the economic burden of a condition that is potentially avoidable; whereas full economic evaluations assess the balance of intervention costs with the resulting health outcomes and avoided costs [34]. If an intervention results in lower total costs as well as better outcomes compared to an alternative strategy it is said to be “dominant” or cost-saving. Relatively few screening programs are net cost-saving [35]. If an intervention results in higher net costs but improves health, it may be cost-effective, depending on the cost per outcome achieved and how much decision makers are willing to pay for improved outcomes. Cost-effectiveness analyses differ in types of outcomes assessed. Cost-consequences analyses only consider intermediate outcomes, for example the cost of screening and diagnostic evaluation per diagnosis achieved. However, “Calculating the cost to detect a case tells one nothing about the value of detecting and treating the disease in question and, hence, is not informative of the balance of costs and outcomes” [36]. In contrast, full cost-effectiveness analyses of screening programs project the long-term health and economic outcomes associated with early identification predicated on various assumptions [37].

The cost-effectiveness of an intervention can only be assessed in comparison to other interventions. Cost-effectiveness analyses may compare UNHS relative to no hearing screening, to a hypothetical selective screening strategy, or to current patterns of care at the time the study was performed. The expected cost-effectiveness of UNHS may differ depending on the assumed alternative to UNHS. In particular, studies that compare UNHS to a hypothetical selective screening that is assumed to identify the majority of infants who are D/HH typically question the efficiency of UNHS. For example, the USPSTF review of 2001 assumed that selective screening would detect 70% as many cases of PCHL as under UNHS and would generate only 20% as many false-positive screening results [23]. Although the USPTF did not assess cost-effectiveness, Kemper and Downs in 2000 similarly projected that selective screening would detect 59% as many cases of PCHL and generate only 5% as many false-positive screening results as under UNHS; the incremental cost per case detected was projected to be eight times greater under UNHS [38]. However, as already noted, although targeted hearing screening based on risk factors was clinically recommended prior to the adoption of UNHS policies in the United States it was very unevenly implemented and few infants were diagnosed as D/HH as a result. The logical comparison for US policy analyses of UNHS was the status quo of limited identification of infants who were D/HH.

Most economic evaluations of newborn hearing screening have been partial analyses that focused on estimating the cost of implementing UNHS using different screening technologies. A smaller number of analyses have attempted to calculate the health and economic benefits of early detection of hearing loss or deafness. In particular, several studies have estimated the potential reduction in costs of special education services for some children who are D/HH associated with early identification and intervention. 

Two systematic reviews of economic assessments of newborn hearing screening strategies were published during 2012. One review article identified 21 papers specific to UNHS that were published in English, French, or German from 2000 through early 2012 [39]. Most were from the United States (*n* = 8) or Germany (*n* = 6). The other systematic review identified 20 studies of UNHS that were published from 1992 through early 2011, of which nine were from the United States and three from the United Kingdom [40]. Although 14 studies were common to the two reviews, 13 were found in just one. Another 12 studies reporting cost estimates published prior to 2012 were cited in neither systematic review.

Given the substantial differences in cost structures among countries, as well as our interest in relating economic evidence to policy decisions in the United States, we focus here on empirical estimates specific to the US context. Readers interested in cost estimates from other countries are encouraged to read the published systematic reviews [39,40].

### 2.1. Costing Studies of US Hospital-Based UNHS Programs

Multiple studies have assessed the cost of hospital-based hearing screening in terms of average cost per infant screened and/or cost per case of hearing loss or deafness detected. Some studies have compared the costs of different UNHS algorithms used to screen newborns in regular maternity units involving the use of either automated auditory brainstem response (AABR) alone or the use of transient evoked otoacoustic emissions (TEOAE) or distortion product otoacoustic emissions (DPOAE) as a first screen. Often, OAE screening is followed by screening using AABR for infants who do not pass the initial screening. The relative merits of OAE, AABR, or two-tier OAE/AABR screening algorithms for hearing screening in well-baby units continue to be debated [41,42]. 

The estimated cost per infant screened is highly variable across studies. Table 1 reports screening cost estimates from 10 US studies which reported micro-costing estimates of hospital-based UNHS, all of which were specific to screening of newborn infants who were not in neonatal intensive care units or special care nurseries. Most of those studies were not included in previously published reviews of UNHS economic evaluations. To increase comparability, only the costs of pre-discharge screening are reported and cost estimates were adjusted for inflation to 2016 US dollars [43]. Unlike previous reviews, studies that reported hypothetical estimates or simply repeated empirical UNHS cost estimates from earlier published research are not listed.

Most of the cost estimates in Table 1 were in the range of approximately $27 to $47 (2016 US dollars) per infant screened. Differences across studies in cost estimates can reflect methodological differences and true cost differences. Hearing screening costs in a given setting can vary as a function of multiple factors, including the type of staff conducting the screening, the hourly wage of screeners, the amount of supervision and administrative oversight required, and the number of infants over whom fixed equipment costs are spread. Several studies provided detailed information on such factors, whereas others did not. 

Of the three studies in Table 1 that compared the costs of different screening technologies or algorithms, one estimated that the use of OAE alone was considerably less expensive than AABR alone. Similarly, an unpublished analysis of UNHS cost data collected by CDC researchers from hospitals in six states in 1997 and presented to the JCIH the same year found average costs of $28.38 for hospitals using OAE alone and $41.13 for those using AABR alone [53]. In contrast, two other studies in Table 1 found the costs to be similar for the two screening methods. Two studies found the cost of an OAE/AABR algorithm to be similar to AABR alone. However, the full cost of UNHS includes the costs of follow-up, post-discharge screening, and diagnostic evaluation, which can add substantially to the per-child screening costs [52]. Because the rate of false-positive screens is higher with OAE screening, average total costs may be higher than for AABR or OAE/AABR algorithms when follow-up and diagnostic costs are included [50,52,54].

### 2.2. US Assessments of Educational Costs Associated with Bilateral Congenital Hearing Loss or Deafness

The impact of UNHS on educational costs for children who are D/HH is a function of three parameters: (1) the percentage of children who are identified early due to UNHS and receive intervention services before 6 months; (2) the additional cost of schooling for children who are D/HH compared to other children in the absence of early identification; and (3) the percentage reduction in average cost of special education services associated with early identification and intervention. This section addresses US estimates of the additional or incremental educational costs associated with bilateral PCHL, summarized in Table 2, with all cost estimates adjusted for inflation to 2016 US dollars.

Mehl and Thomson reported that in Colorado the mean incremental cost of schooling for children with bilateral PCHL was reported as $6041 in 1993 [55]. That estimate was based on the distribution of schooling placements for Colorado children with hearing loss or deafness listed as the reason for receipt of special education: 13% in special or residential schools, 23% in self-contained classrooms, 28% in resource programs, and 34% in consultative programs, along with estimates of the additional costs for each placement type relative to children not receiving special educational services. Incremental annual costs (in 1993 US dollars) varied by setting from $25,000 for residential schooling, $8300 for self-contained classrooms, $2300 for resource programs, and $700 for consultative/itinerant programs.

The US Department of Education funded a nationwide Special Education Expenditure Project that calculated estimates of the incremental education cost for children receiving special education services during the 1999–2000 school year stratified by “exceptionality” or category of special education services [56]. Those estimates were used in a CDC publication on the economic costs associated with intellectual disability, cerebral palsy, prelingual hearing loss, and congenital vision impairment [59]. Average educational expenditures for children receiving services for hearing loss or deafness alone (The official term for that special education exceptionality at the time of data collection was Hearing Impairment.) were higher by US$9436 per year in 2000 dollars relative to children receiving no special education services [57]. Average expenditures for children with other exceptionalities, some of whom are D/HH but have other qualifying conditions such as autism or intellectual disability, are often considerably higher [56]. Using a 3% discount rate, which has been recommended for US cost-effectiveness analyses since 1996 [60], the present value of educational costs attributable to bilateral hearing loss or deafness was reported to be approximately US$115,600 in 2007 dollars [57]. That estimate presumes that the use of special education services by children who are D/HH is constant from age 3 through 17 years.

Mohr et al. estimated the discounted lifetime extra cost of schooling for children with prelingual (onset at ages 0–2 years) severe or profound bilateral hearing loss or deafness (70 dB or greater) to be $504,900 in 1998 US dollars using a 3% annual discount rate [58]. Children with severe to profound hearing loss or deafness account for approximately 45% of children with bilateral PCHL [28]. Mohr et al. estimated incremental annual schooling costs of $18,645 per child ages 3–17, which was 2.7 times higher than estimated by Mehl and Thomson for Colorado children when expressed in dollars of the same purchasing power (Table 2). That discrepancy in large part reflects a much larger percentage of children educated in high-cost special schools, 42% in Mohr et al. vs. 13% in Mehl and Thomson; the latter estimate is population-based and reflects official statistics in the state of Colorado in 1993. In comparison, Mohr et al. used information on special education placements from the US Department of Education Office of Special Education and Rehabilitation Services 1997 annual report for 63,000 children who were D/HH and some 1000 children who were deaf/blind; Mohr et al. chose to use information only on the children classified as deaf/blind [58]. In addition, the high lifetime cost estimate in Mohr et al. appears to be due at least in part to a calculation error. Taking an annual incremental education cost of $18,645 from ages 3 through 17 years and applying a 3% discount rate, the present value in 1998 dollars can be calculated to be $208,000 versus the $504,900 reported.

### 2.3. US Cost-Effectiveness, Cost-Benefit, and Cost-Savings Analyses

Several published studies have estimated projected education cost savings associated with UNHS/EHDI in the United States along with estimates of screening and diagnostic costs; some of the studies also included additional cost types and outcome measures. Characteristics of the included studies are summarized in Table 3. 

Mehl and Thomson [55], in 1998, published the first US assessment of the potential economic benefit or cost-savings associated with EHDI. That study projected that EHDI would result on average in 37% lower special education costs. Specifically, those authors assumed that timely diagnosis and intervention would lead to shifts in the distribution of children who are D/HH receiving special education services by setting, with one-half of children requiring less resource-intensive services (or none) (see Section 2.2). In addition, it was assumed that half of children who are D/HH would no longer require 2 years of preschool special services. Overall, reductions in educational costs as well as in speech therapy were projected to exceed the cost of UNHS within 12 years [55]. That study optimistically assumed that all children with PCHL would be diagnosed and receive services early, with no loss to follow-up. However, many US children who do not pass UNHS are not documented to receive a diagnostic evaluation, and among those who are diagnosed as D/HH many do not start receiving intervention services by 6 months of age, although there has been progress over time in both measures [32,33,62,63]. 

The first formal US economic analysis of UNHS relative to an alternative was a cost-consequences analysis published in 2000 that compared short-term costs and projected numbers of diagnoses for UNHS and targeted screening strategies [38]. Kemper and Downs assumed that UNHS and targeted screening would respectively detect 86 and 51 cases of “congenital deafness” per 100,000 infants screened. On that basis, the authors projected that the cost per child identified would be 3.7 times greater with UNHS than under targeted screening. However, in practice, relatively few children who were D/HH were identified through targeted hearing screening protocols that were based on risk factors present at birth [5,6,64,65]. A cost-consequences analysis by Kezirian and White calculated the average cost per case identified in UNHS compared to no screening for two different algorithms [49]. 

Keren et al. in 2002 also compared the cost and diagnostic yield of hypothetical UNHS and targeted screening strategies, adopting assumptions similar to Kemper and Downs and reaching similar conclusions about the greater theoretical efficiency of targeted screening [51]. In addition, Keren et al. also conducted a full cost-effectiveness analysis that projected the long-term effects of early detection of PCHL on medical and audiological costs, education costs, and employment and earnings [51]. They assumed that 50–70% of children who received intervention by 12 months of age would have language development in the typical range, compared with 28–40% of those identified later. Keren et al. also assumed that children who developed typical language would experience a 10% reduction in excess educational costs and a 75% reduction in lost productivity. Adjusting the Mohr et al. estimates of lifetime education costs and lost productivity for inflation to US$550,000 and $433,000, respectively, in 2001 US dollars, Keren et al. projected that screening would quickly yield net economic benefit [51]. However, the Mohr et al. estimates of both educational costs and productivity costs were calculated for persons who were classified as deaf/blind [58] and do not apply to a general population with moderate to profound bilateral PCHL. Further, the assumption that 75% of the gap in earnings with adults who have typical hearing would be eliminated was extremely optimistic, as noted by Colgan et al. [40].

Similarly, Gorga and Neely in 2003 [61] assumed a US$1,000,000 lifetime present value cost of PCHL, which included both higher education costs and lower lifetime earnings (productivity costs) taken from Mohr et al. [58]. In addition, Gorga and Neely assumed that EHDI would result in a 100% reduction in costs associated with PCHL [61]. On the basis of that extremely optimistic assumption, Gorga and Neely projected that EHDI would result in a net economic benefit after 4 years, assuming that in the absence of UNHS, no infants would be screened for hearing loss or deafness. The assumption that EHDI would avert 100% of the educational costs associated with PCHL is highly implausible; the challenge is to find an empirical estimate of the proportionate reduction in educational spending. Like most studies other than Keren et al. [51], Gorga and Neely did not take into account the costs of screening follow-up, diagnosis, and early intervention services [61].

To produce accurate estimates of avoided costs with EHDI, researchers can use real-world estimates of effectiveness comparing cohorts who were or were not exposed to UNHS/EHDI. In 2007, Grosse estimated the aggregate potential averted educational costs associated with EHDI in the US by combining US estimates of the extra cost of education for children with moderate or greater bilateral PCHL with published UK estimates of the relative reduction in educational costs associated with early identification [57]. When children in the UK Hearing Outcomes Project were evaluated at 7–8 years of age, UNHS was associated with a 36% reduction in incremental education costs associated with hearing loss or deafness, or a 22% reduction in gross costs of schooling [66,67]. Grosse applied the 36% percentage reduction in extra schooling costs to the estimated lifetime extra cost of education of $115,600 in 2007 US dollars [57] (see Section 2.2). The result was equivalent to a reduced cost of $44,200 per child detected at birth through UNHS who received intervention services. Further, it was assumed that 1.2 per 1000 children with moderate to profound bilateral PCHL are detected through EHDI programs and receive intervention services. Given 4 million births per year, an estimated 4800 children would benefit each year. Multiplying by $44,200 results in an estimated educational cost offset of $212 million each year in 2007 US dollars, or $53 per newborn. Grosse noted that this was similar to published estimates of the per-child cost of UNHS, although no attempt was made to estimate the cost of additional EHDI infrastructure or programmatic expenditures, including specifically the cost of providing intervention services [57]. Similarly, the study did not take into account loss to follow-up for diagnostic and intervention services.

## 3. Discussion

### 3.1. The Role of Economic Evidence in the Evolution of EHDI Policy in the United States

The United States has a decentralized approach to health policy. Professional societies and advisory bodies, such as the American Academy of Pediatrics and the USPSTF, make recommendations about what preventive health care services should be provided, but government agencies (in the case of publicly funded health care) or individual providers make decisions about recommending or offering those services. Finally, federal and state governments decide whether or not to fund public health programs to support and monitor the delivery of specific preventive services. Cost-effectiveness is not commonly used as a criterion for policy decisions in public health in the United States [68]. That is true specifically for newborn screening policies [69], with a few exceptions [70]. It should also be noted that most policy decisions for other types of newborn screening in European countries likewise have not been informed by cost-effectiveness analyses, although screening costs are often considered [71,72]. Similarly, it is common for US policy decisions on newborn screening to have been informed by estimates of screening costs [69].

As noted in the first section, the development of new screening methods policies and the publication of findings in the late 1990s indicating that D/HH children identified earlier typically had better language outcomes led to growing professional and governmental support for UNHS [16,20]. The adoption of UNHS legislation by a growing number of states and funding for EHDI programs provided by the US Congress through HHS agencies led to the rapid implementation of UNHS/EHDI across the United States. It is notable that the first evidence-based recommendation by the USPSTF in support of UNHS was released in 2008 [29], long after hearing screening had become universal throughout the country. One reason for that delay was that advocates of evidence-based practice were focused on the theoretical advantages of the greater efficiency of selective screening of high-risk infants [23,27,38], even though such screening was not commonly practiced in the United States and might not have been feasible.

The primary impetus for the adoption of legislation in Colorado in 1997 was the successful implementation of the CNHS project and the findings of research conducted by researchers from the University of Colorado Boulder that demonstrated better outcomes with earlier identification and intervention [17]. Yoshinaga-Itano et al. found that children who were D/HH and were identified prior to 6 months of age had statistically significantly better language outcomes than children who were diagnosed later [3]. Although that research was published in peer-reviewed form in 1998, the findings had already informed the policy development process in Colorado and elsewhere. Similarly, an evaluation of CNHS project data found that 84% of children who were D/HH born in participating hospitals were identified with hearing loss or deafness prior to 6 months of age, compared with 8% of children who were D/HH born in other hospitals. Moreover, the average language quotient was 82 for the UNHS hospital group and 62 for the non-UNHS hospital group [18].

Formal cost-effectiveness analysis does not appear to have been an important direct contributor to the EHDI policy process in the United States. On the other hand, stakeholders are often interested in understanding the cost or budgetary implications of new programs. That demand was addressed by US cost analyses of hospital-based UNHS that appeared between 1995 and 2002 and are summarized in Table 1. Those studies indicated that the resource cost of screening was modest by hospital standards, $52 or less per newborn infant in 2016 US dollars, with most estimates in the $27–47 range.

However, even if cost-effectiveness is not an official policy criterion, economic assessments which project that an intervention may pay for itself in terms of avoided direct costs may be influential. For example, the informal economic assessment by Mehl and Thomson [55] informed the policy development process in the state of Colorado. That study used Colorado education records and projected that reduced costs of special education services could plausibly offset the cost of UNHS within a space of 10 years. The findings of that analysis allayed concerns from some legislators of the cost implications of the new policy. Mehl and Thomson’s calculations assumed a 36% reduction in special education costs, which coincides with UK estimates published in 2006 [66,67]. Extrapolating from that relative reduction, Grosse projected that that reduction in costs, if sustained over time and assuming prompt diagnosis and timely receipt of intervention services, could fully offset the cost of UNHS [57]. Two subsequent economic evaluations by Keren et al. [51] and Gorga and Neely [61] likewise projected potential cost savings of UNHS in comparison with no screening. However, those studies relied on very optimistic assumptions of reductions in costs. Prospective economic assessments often employ optimistic assumptions. On the other hand, if one waits for the availability of evidence of long-term outcomes to conduct economic evaluations, the opportunity to inform policy decisions may be missed.

### 3.2. Future Economic Evaluations of UNHS/EHDI Programs and Policies

Population-based empirical estimates of long-term effectiveness and costs are crucial to robust retrospective economic evaluations. Although the effectiveness of UNHS/EHDI in terms of language outcomes is contingent on timely diagnosis and initiation of intervention services [73], the proportions of infants who are D/HH who receive timely identification and intervention services have generally been left out of economic evaluations. Economic assessments of EHDI have also typically not included the follow-up costs of diagnostic and intervention services in addition to the cost of initial screenings. It would be appropriate for future economic assessments of EHDI policies to include not only those clinical services but also the costs of public health EHDI programs, which includes the costs of developing and maintaining data systems for monitoring and supporting follow-up services. 

Economic assessments of the benefits of EHDI could also provide more accurate estimates if they were able to include data on multiple language outcomes, educational expenditures, and labor market outcomes associated with differences in schooling and language outcomes. It would be helpful to have information on educational expenditures for students who are D/HH stratified by the presence of other conditions and by the setting in which services are received, e.g., schools for the Deaf. Economic studies could also calculate costs that may be incurred by the healthcare and criminal justice systems associated with differences in schooling and language outcomes among people who are D/HH. Although previous economic assessments have demonstrated reduced educational expenditures as well as better outcomes, additional evidence could confirm and refine estimates of the level of benefits resulting from EHDI. Finally, future cost-effectiveness analyses might assess how EHDI is associated with differences in physical, mental, and emotional health outcomes, both overall and among subgroups stratified by language acquisition type and sociodemographic indicators in order to assess implications for disparities.

Additional analyses of educational records could further assess the extent to which EHDI has resulted in improved language outcomes and reduced educational expenditures for children who are D/HH. A recently published US study found that children who were D/HH ages 9–39 months who met all three components of the EHDI guidelines (hearing screening by 1 month, diagnosis of hearing loss or deafness by 3 months, and intervention by 6 months of age) had significantly better vocabulary quotients [73]. A natural experiment in two Australian states found that for children who were D/HH without intellectual disability, UNHS was associated with significantly better receptive and expressive language and receptive vocabulary [74]. Finally, a recent publication from the UK of a follow-up cohort of 73 adolescents with PCHL included in a previous trial of UNHS [11,28] found that 61% (41–78%) of those born during UNHS periods were educated in mainstream schools versus 39% (22–59%) of those born during periods without UNHS; differences in education costs were not reported [75].

## 4. Conclusions

This review has assessed the role that economic arguments played in the widespread adoption of UNHS policies in the United States between 1996 and 2006. While it does not appear that formal cost-effectiveness analyses influenced the policy process during that period, there is evidence that the results of both cost assessments and informal assessments of the potential benefits in terms of reduced spending on special education services were considered and may have influenced the adoption of UNHS policies. That conclusion is consistent with the experience of the limited role of formal economic assessments in newborn screening policies in most of the United States as well as in the majority of European countries. Although formal economic analyses can provide valuable information for decision makers, depending on the accuracy of the assumptions made, even partial or informal economic assessments may provide useful information that can advance the development of UNHS policies.

## Figures and Tables

**Table 1:** US micro-costing studies estimating inflation-adjusted cost (in 2016 US$) of pre-discharge hospital-based newborn hearing screening in well-baby units, by type of screening algorithm (does not include post-discharge testing or diagnostic costs).

Study ^	OAE	AABR	OAE/AABR
Downs (1995) [17]			$47.08
Maxon et al. (1995) [44]			$46.71
Barsky-Firkser and Sun (1997) [45]		$51.55	
Weirather et al. (1997) [46]	$12.40		
Mason and Herrman (1998) [47]		$35.49	
Gorga et al. (2001) [48]			$28.02
Kezirian et al. (2001) [49]	$20.28	$32.18	$31.72
Vohr et al. (2001) [50]	$45.08	$51.55	$51.93
Keren et al. (2002) [51]			$27.35
Lemons et al. (2002) [52]	$49.44	$51.66	

^ Studies arranged in chronological order; US cost estimates from different years adjusted to 2016 US dollars using the Personal Consumption Expenditure Health Care by Function Index, Bureau of Economic Analysis [43] OAE—Otoacoustic Emissions; AABR—automated auditory brainstem response.

**Table 2:** Estimated annual and/or discounted present value of incremental costs of special education associated with permanent congenital hearing loss in US children, 2016 US dollars.

Study	Data Source	Annual Cost	Present Value
Mehl & Thomson [55]	Colorado Department of Public Health & Environment	$9308	
Chambers [56]	U.S. Department of Education, Office of Special Education Programs, Special Education Expenditures Project	$12,389	
Grosse [57]	Analysis of Special Education Expenditures Project data [56]		$132,320
Mohr et al. [58]	US Department of Education, Office of Special Education and Rehabilitation Services, 1997 annual report, data on children who were deaf/blind who had onset before age 3 years	$26,318	$712,681

US cost estimates from different years adjusted to 2016 US dollars using the Gross Domestic Price deflator, US Bureau of Economic Analysis. Calculated using 3% annual discount rate.

**Table 3:** Published partial or full US economic evaluations of UNHS/EHDI policies or programs.

Study	Study Type	Intervention Costs	Outcome Measures	Key Assumptions on Outcomes
Mehl & Thomson [55]	Potential cost-savings	Screening	Avoided excess cost of education	37% reduction in excess cost of education
Kemper & Downs [38]	Cost-consequences analysis	Screening	Number of cases identified	Not applicable
Kezirian & White [49]	Cost-consequences analysis	Screening	Number of cases identified	Not applicable
Keren et al. [51]	Cost-consequences and cost-effectiveness analyses	Screening, diagnosis, and intervention	Number of cases identified, avoided excess cost of education, increased labor productivity	Children who have “normal“ language have 10% lower excess cost of education and 75% lower productivity losses
Gorga & Neely [61]	Potential cost-savings	Screening	Avoided excess cost of education, increased labor productivity	100% reduction in excess education costs and productivity costs
Grosse [57]	Potential cost-savings	Screening	Avoided excess cost of education	36% reduction in excess cost of education
